# Expression of Basic Emotions in Pictures by German and Vietnamese Art Therapy Students – A Comparative, Explorative Study

**DOI:** 10.3389/fpsyg.2019.00975

**Published:** 2019-06-19

**Authors:** Alexandra Danner-Weinberger, Katharina Puchner, Margrit Keckeis, Julia Brielmann, Minh Thuy Thi Tri, The Huy Le Hoang, Luan Huynh Nguyen, Nikolai Köppelmann, Edit Rottler, Harald Gündel, Jörn von Wietersheim

**Affiliations:** ^1^Department of Psychosomatic Medicine and Psychotherapy, Ulm University Medical Center, Ulm, Germany; ^2^Faculty of Psychology, University of Social Sciences and Humanities, Vietnam National University Ho Chi Minh City, Ho Chi Minh City, Vietnam

**Keywords:** art therapy, intercultural comparisons, Asian and Western culture, self-therapy, self-experience

## Abstract

During art therapy self-experience workshops in Vietnam and Germany, the authors noticed that there were differences in how the groups expressed their feelings in paintings. This led the authors to a comparative, explorative study. In this study workshop participants from Germany and Vietnam (29 in each group) were instructed to draw pictures related to basic emotions like sadness, disgust, anger, or happiness. Then, the paintings were evaluated by using rating scales with which formal and content-oriented criteria can be assessed. The interrater reliability was good. The results showed some statistically significant differences. The Vietnamese participants used smaller formats and more colors with stronger color application. They preferred simpler forms than the Germans. The German participants painted in a more abstract manner, while the Vietnamese participants used more concrete images and tended to scenic expressions (representation of stories). There are some reasons which might explain these differences: It is known that cultural influences play an important role in the arts as well as in the way creative arts are taught in a particular society. The expression of emotions is culture-specific as well. The Vietnamese participants were, however, interested in the possibilities art therapy offers and felt that there are good chances for this therapy to develop in their country.

## Introduction

Based on a clinical-scientific partnership with Vietnam and initiated by the Department of Psychosomatic Medicine and Psychotherapy of the University Hospital of Freiburg, workshops lasting several days were held in Hanoi in December 2013 and December 2014 (Fritzsche et al., 2008, 2014). The goal of these workshops was to get to know each other and discuss clinical pictures, research and psychosomatic and psychotherapeutic treatment options in Germany and Vietnam. In December 2014, as part of the 2nd Vietnamese - German Scientific Conference on Psychosomatic Medicine and Psychotherapy, we presented art therapy during a workshop for the first time as a creative, therapeutic method and found that there was a great deal of interest from the participating Vietnamese psychologists and physicians. From this interest, a continuing education course in art therapy in Ho Chi Minh City was then developed. Several visits and more workshops followed.

During the first experiential self-experience exercises, we had the impression that the Vietnamese participants expressed feelings in pictures quite differently than the German art therapy students with which we had experience up to that point. Their paintings were more precise and recounted more episodes from the respective life stories. These initial clinical impressions then led to the research project described here.

In Germany, art therapy has been established as an independent therapy for a long time. There are several private and public universities in which art therapy is offered as continuing education or even as a course of study (Deutscher Fachverband Kunst- und Gestaltungstherapie, 2018). In Vietnam, a country in which the healthcare system is developing slowly and where there are few financial resources available, there has not been any art therapy up to that point as far as we know. In areas of Asia that are economically better off such as South Korea and Japan, art therapy is known as a method of treatment, and there are corresponding courses of study for art therapists (Kalmanowitz et al., 2012).

An important component of learning a psychotherapeutic method, including art therapy, is personal therapy (Orlinsky et al., 2011). The goal is for participants in the training to experience the effects of the method personally and to thereby also become familiar with the possibilities and limits of the method. In addition, they learn therapeutic techniques and can work on their own blind spots and the development of their personality. Art therapy requires greater self-experience and personal experience as well. This is why the first step in continuing education classes in Germany and Vietnam was to ask participants to draw pictures themselves and then to talk about the pictures and the processes as well as their emotional experience in a group setting.

In the development of mental disorders and their therapeutic treatment, emotions are an important component in almost every method. Inner tensions and conflicts are expressed in emotions, and mental conflicts and illnesses change emotionality. It was, therefore, a good start for the planned group experience to have basic emotions and events in which these were experienced drawn in pictures.

There is some debate in how far emotions are universal, like biologic reactions, and in how far they are culture specific. According to Ekman (Ekman, 1972; Ekman and Cordaro, 2011), for the so-called basic emotions, it is assumed that these are present in everyone, regardless of culture, and they are experienced in a similar manner. On the other hand, coming from a linguistic perspective, Russell (1991) states that there are similarities between emotions in different countries, but they are not identical. So, there are differences between cultures in the number of emotional words. In some cultures, words for special emotions are missing, or there are special emotions, which are not known in all cultures. Also, the emotions, which are related to an event (e.g., separation or divorce) like anger, sadness, shame, might be very different between cultures. This debate is still ongoing (Gendron et al., 2015). Newer research including brain studies shows how these approaches can be combined (Barrett, 2017).

Some of the possible causes for a different expression in the context of art-therapeutic self-experience exercises are cultural factors, a different way of dealing with emotions in society, a different meaning of colors, different experiences in dealing with artistic creativity, and may also include a different willingness to express emotions as part of a self-experience exercise. Different family systems, a strong family connection with several generations in close living quarters in Vietnam, and different economic and political conditions may have an influence here as well.

Such intercultural differences must be reflected when doing these studies. For example, when constructing the Japanese version of the Levels of Emotional Awareness Scale (LEAS-J), it came out that original (US-American) scenes, which should provoke emotions, had to be changed because they would not be understood by the Japanese participants or would not lead to the wished emotions. Also, norm values between Japanese and US-American students were different: In Japanese participants, there was a higher social desirability and less emotional expression in the society (Igarashi et al., 2011).

Intercultural aspects have also been discussed in art therapy and art therapy research. Betts (2013a) points out that in assessment tools, the equivalence of the instruments in different cultures has to be checked carefully. Using examples of standardized instruments, she shows how cultural aspects have an influence on the results of assessments (Betts, 2013b).

The main objective of this project was to review the therapeutic impressions that we made on the basis of our first practical experiences. We had the impression that the way Vietnamese students describe emotions in paintings were somewhat different to those of German students. To this purpose, the following explorative sets of questions were developed:

Which formal and content-related commonalities and differences can be seen between Vietnamese and German pictures of emotions regarding

(a) formal criteria such as material, colors, sheet size and sheet use?
(b) content-related criteria such as the subject of the picture, abstraction, use of symbols?

## Materials and Methods

### Creation of the Pictures

The pictures were created during the art therapy self-experience workshop offered as part of continuing art therapy classes in Germany and Vietnam. They were taught by the same trainer (ADW) and held in Ho Chi Minh City in Vietnam in December 2015 and November 2016 as well as in Germany in March 2016 in Ulm and in October 2016 in Munich.

The objective was to draw basic emotions according to Ekman (1972) in a standardized art therapy setting. Ekman lists the following basic emotions: fear, distrust, happiness, sadness, surprise, and anger. The procedure in all courses was identical. During a 3-day training, participants in a group of 13–20 persons were instructed as follows: “Think of a situation you maybe experienced yourself, a situation that a patient has described, or a fictitious situation from a movie/book/fairy tale, etc., in which for example the emotion anger (one of the six basic emotions) is experienced. Draw a picture about this emotion.” Then, participants were given 45–60 min creative time, which was followed by a group discussion of the pictures. All pictures were photographed with a digital camera and stored in a computer as an image file. With 58 participant and 6 basic emotions, a total of 348 pictures were created.

All groups were provided with the following materials in the same form for the creation of the pictures: pencil, charcoal, colored pencils and markers, wax crayons (normal and water-soluble), pastel chalks, watercolor, liquid paint (acrylic, tempera), string, tape, glitter, white paper in sizes A2, A3, A4, brown (packing) paper in sizes A3 and A4, colored cardboard and tissue paper.

### Sample

The pictures were created by a total of 58 training participants, 29 from Vietnam and 29 from Germany. All participants had either completed therapeutic, pedagogical or social-pedagogical professional training, and most of them did not have any prior experience with art therapy. If anything, the participants were at the beginning of their art therapy training. All German participants were women. They ranged in age from 30 to 58. In the Vietnamese group, there were 7 women and 22 men ranging in age from 25 to 45.

The participants gave their informed and written consent to the participation in the study and allowed us to evaluate the paintings in this study. According to the rules of the Ethics Committee of Ulm University and national rules, a formal ethic vote was not necessary: It is research on data of adult and healthy probands on a completely anonymized data set. No individual-related data were assessed and stored and there was no clinical intervention. The pictures were stored and managed in compliance with the data protection provisions.

### Evaluations

The analysis instrument used for the pictures was the rating form by Poetsch (2010), published in Oster et al. (2013) in a slightly modified version. This form permits a systematic and reliable documentation of formal and content-related aspects of paintings. The form as used consists of 18 items (refer to Table 1). The items have specific response categories. The form documents formal criteria such as color, color application, the size of the picture, and also content-related criteria (such as the use of symbols and the level of abstractness or concreteness of the picture). In deviation from the previous version, it was specified for the coding of the colors that only those colors should be coded that cover at least 20% of the area. This made it possible to code up to 5 colors simultaneously (all colors below 20% were rated as “multi-colored.”

**TABLE 1:** Rating scale for the picture analysis (according to Oster et al., 2013).

**Criterion**	**Choices (C)**
(1) Predominant color (quantitative)	Up to five colors that covered at least 20% of the painted area are coded. If there were more, the code “multi-colored” was used. A total of 12 colors (choices) were provided
(2) Number of colors	1–4/5–7/>7
(3) Color application	Thin/thick/mixed/no color application
(4) Color quality	Bright/subdued/mixed
(5) Material used	9 Choices, multiple answer (such as pencil, colored pencil, markers, watercolor)
(6) Techniques used	Multiple answer: Drawing/painting/spatial techniques
(7) Predominant shape	Round/angular/mixed/not definable
(8) Form variety	Simple/many or complicated/not definable
(9) Form quality	Delimited/blurred/mixed
(10) Form size	Big/small/big and small/not definable
(11) Spatial effect	Strong/some/none
(12) Use of the page	Full/almost full/partial/very little
(13) Format used	(a) A2/A3/A4/different format (b) Portrait/landscape/free format
(14) Paper used	White/other (multiple answers)
(15) Concreteness	Predominantly concrete/predominantly abstract/both
(16) Depiction of a human	Entire body/body parts (3 choices)/none
(17) Writing/frequent symbols	Multiple answer, 8 choices: such as Writing, Heart, Tree, Flower…
(18) Expression	Predominantly figurative/predominantly abstract/not clear

Because of organizational reasons, the study took place in two parts. In every part, one rater evaluated all paintings, and a second rater, as a control, rated about a third of the paintings. (First part: rater KP, control rating by MK, second part: all ratings by MK, control ratings by JB). The raters were art therapists, who had advanced art therapy training or who had already completed their art therapy training. They evaluated the pictures on the basis of the rating form referred to above. The rating was performed by emotions (e.g., only pictures on sadness) and was blind, i.e., the raters did not know whether the picture was from Germany or from Vietnam. The raters were provided with the picture in the form of a picture file and were, at the same time, provided with information about the format of the pictures. The rater then looked at the picture on the computer screen and evaluated it on the basis of the rating form. The second rater evaluated 125 of the total 458 pictures (35.9%) independently from the first rating. An interrater analysis showed high correlations. The values for Cohen’s Kappa for 85.7% of the items were above 0.60; often, however, significantly higher (38% above 0.80). Lower Kappa values (for example regarding the items color application, use of a colored pencil) might have been due to the fact that the raters were supposed to evaluate the pictures not in the original but as a picture file. This could result in some uncertainties. When there were rating discrepancies, a consensus rating was built after a discussion between the two raters, which was then included in the group comparisons.

### Statistical Evaluations

The ratings of the pictures were then entered into an Excel file and evaluated with SPSS. The variable that was used for the interrater correlation was Cohen’s Kappa for categorical data. The significance test used to compare the two groups from Vietnam and Germany was the Chi^2^ test or respectively the exact Fisher test. *P*-values below 0.05 are interpreted as significant. No Alpha adjustment was made because this is an explorative study. This is in accordance with Rubin (2017) who states that Alpha adjustment is less necessary in explorative studies if the test themselves are independent. This was the case here.

## Results

Based on the 18 items in the rating forms, comparisons were made between the Vietnamese and the German participants. There were no significant differences regarding the variables “Spatial effect of the pictures,” “techniques used (drawing, painting).”

Regarding the formal criteria, there were many significant differences, which are summarized in Table 2: The German participants significantly preferred larger formats (DIN-A2). The Vietnamese participants preferred smaller formats (DIN-A3). The Vietnamese participants used significantly more colors, and their color application was stronger. They used more clear and delimited shapes, while the shapes of the German participants were rather blurred. The Vietnamese participants preferred simpler shapes, while Germans often drew an undefinable shape. There were also statistically significant differences regarding the material used: The Vietnamese primarily and much more than the Germans used colored pencils and liquid paints, while Germans seemed to prefer wax crayons and watercolor (refer to Table 3).

**TABLE 2:** Formal criteria (percentages per country, counted per group *N* = 174).

**Item**	**Vietnamese Participants Ratings/Percentages**				**German Participants Ratings/Percentages**				***p***
Page size (DIN)	A2	A3	A4	Other	A2	A3	A4	Other	
%	2	69	28	1	22	51	25	1	<0.001

Number of colors	1–4	5–7	>7		1–4	5–7	>7		
%	32	41	27		57	29	14		<0.001

Color application (predominant)	Thin	Thick	Both		Thin	Thick	Both		
%	10	71	19		24	52	24		<0.001

Form quality	Clearly delimited	Blurred	Both		Clearly delimited	Blurred	Both		
%	53	17	30		26	58	16		<0.001

Shape diversity	Simple	Complicated	Not identifiable		Simple	Complicated	Not identifiable		
%	58	22	20		43	10	47		<0.001

Predominant shape	Round	Angular	Both	Unclear	Round	Angular	Both	Unclear	
%	22	22	29	26	26	14	14	47	<0.001

**TABLE 3:** Use of materials (in percent).

	**Vietnamese participants**	**German participants**	***p* (Chi2 test)**
Pencil	11	19	
Color pencil	20	9	<0.010
Wax crayons	31	41	
Marker	20	13	
Pastel chalk	7	10	
Watercolor	32	40	
Liquid paint	71	42	<0.001
Glitter	4	1	
Objects	25	3	<0.001

There were a number of significant differences regarding the content-based criteria as well, which are illustrated in Table 4: While the Vietnamese participants tended to use scenic expression (representation in episodes, stories), the German participants preferred gestures and intimations. Consequently, the content of the Vietnamese pictures was more concrete, while the content of the German pictures was more abstract. Bodies and body parts were drawn relatively infrequently in both groups. They can be seen in 33% of the Vietnamese pictures and in 17% of the German pictures.

**TABLE 4:** Content-related criteria (percentages per country, counted per group *N* = 174).

**Item**	**Vietnamese participants**			****	**German participants**			****	***p***
Expression (predominant)	Scenic	Gestural	Unclear		Scenic	Gestural	Unclear		
	43	34	23		23	61	16		<0.001

Picture content, concreteness (predominant)	Concrete	Abstract	Both		Concrete	Abstract	Both		
	45	37	18		26	65	9		<0.001

Picture shows people	None	Entire body	Faces/smileys	Other body parts	None	Entire body	Faces/smileys	Other body parts	
	67	17	8	8	83	6	8	3	<0.010

A clear and statistically significant difference can be seen in the use of symbols as well: 51% of the Vietnamese and 29% of the German participants used at least 1 symbol (*p* < 0.001). The most common symbols were flowers, hearts and trees (refer to Table 5).

**TABLE 5:** Use of writing and symbols (in percent).

	**Vietnamese participants**	**German participants**	***p* (Chi^2^ test)**
Writing	17	12	
Flower	18	6	<0.010
Heart	12	3	<0.010
Tree	11	5	
Animal	8	8	
Cross	4	2	
House	3	0	<0.050
Question mark	3	1	
Uses at least one symbol	51	29	<0.001

**FIGURE 1:**
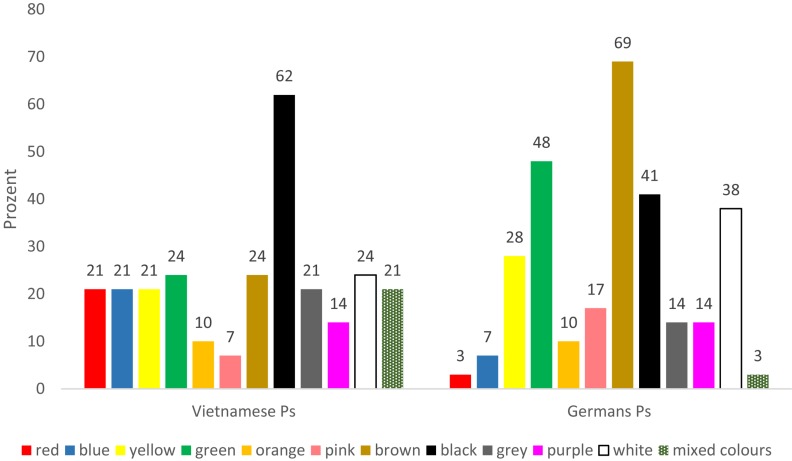
Emotion disgust, colors in percent. *N* = 29 per country.

**FIGURE 2:**
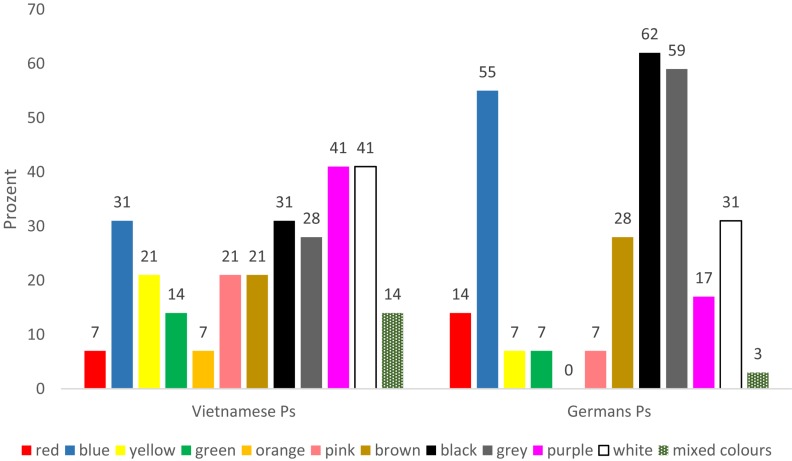
Emotion sadness, colors in percent. *N* = 29 per country.

Regarding the use of the colors, there were only a few differences when considering all pictures and all basic emotions together in the evaluation: The German participants slightly preferred black and brown colors, the Vietnamese participants preferred white and multi-colored (several colors below 20%). Significantly more differences were found when comparing pictures showing the same basic emotion. In the pictures showing disgust, for example, the Vietnamese participants used much more black and red colors, while the German participants used much more brown and green (refer to Figure 1). When expressing sadness, Germans used significantly more black, gray, and blue, while the Vietnamese used much more purple and white (refer to Figure 2). This seems to be a reflection of probably cultural associations between colors and these emotions.

**FIGURE 3:**
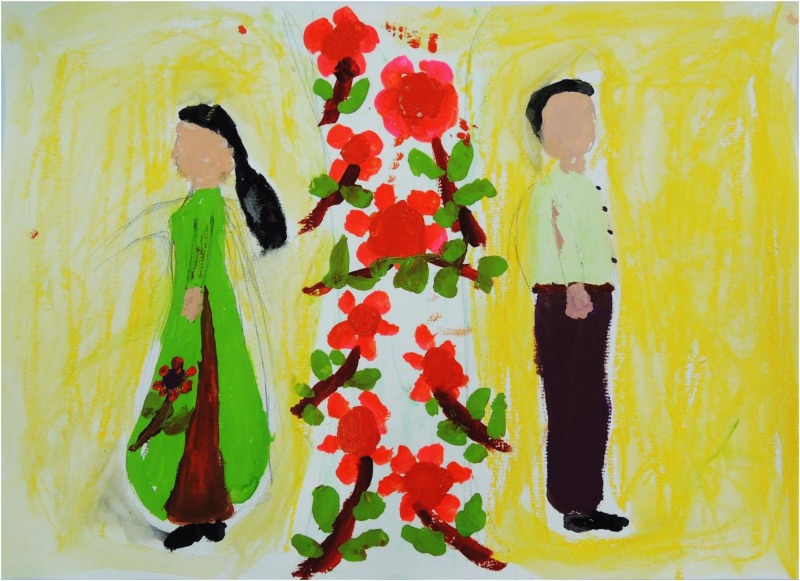
Sadness, painted by a Vietnamese participant.

**FIGURE 4:**
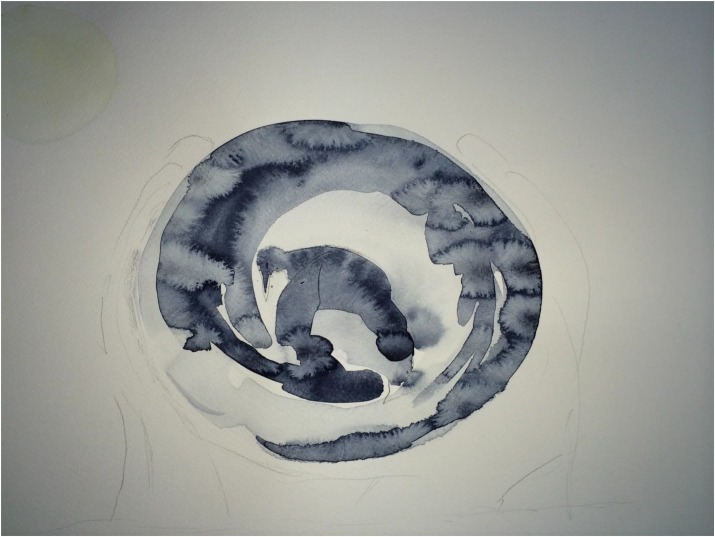
Sadness, painted by a German participant.

**FIGURE 5:**
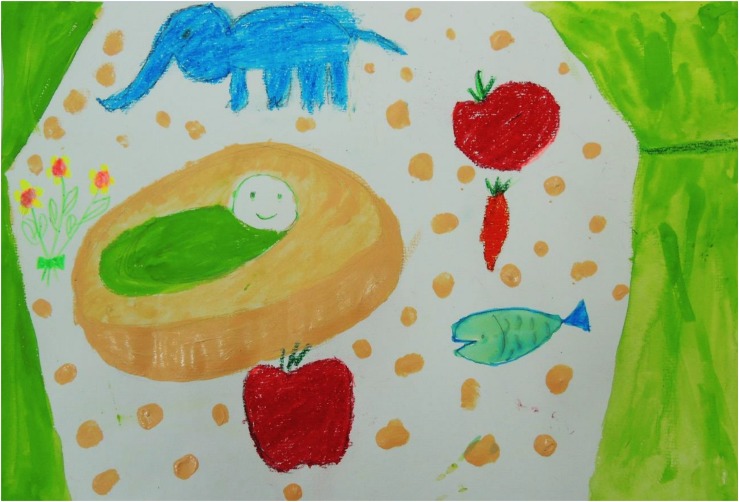
Happiness, painted by a Vietnamese participant.

**FIGURE 6:**
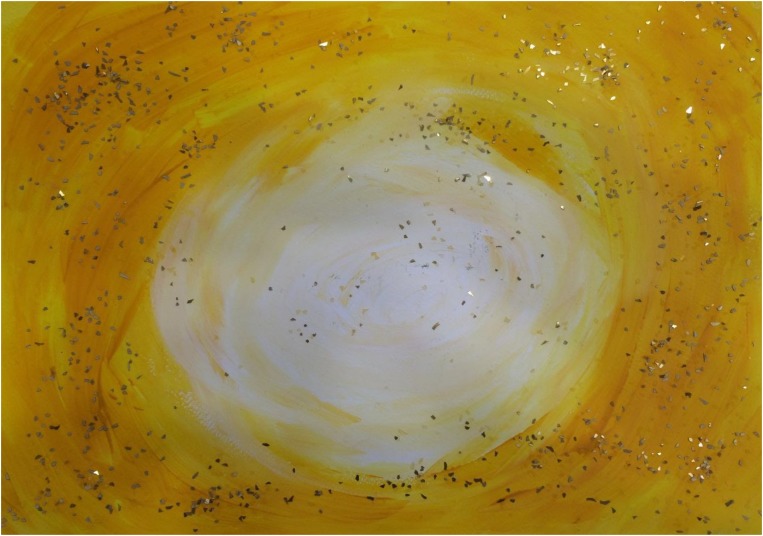
Happiness, painted by a German participant.

**FIGURE 7:**
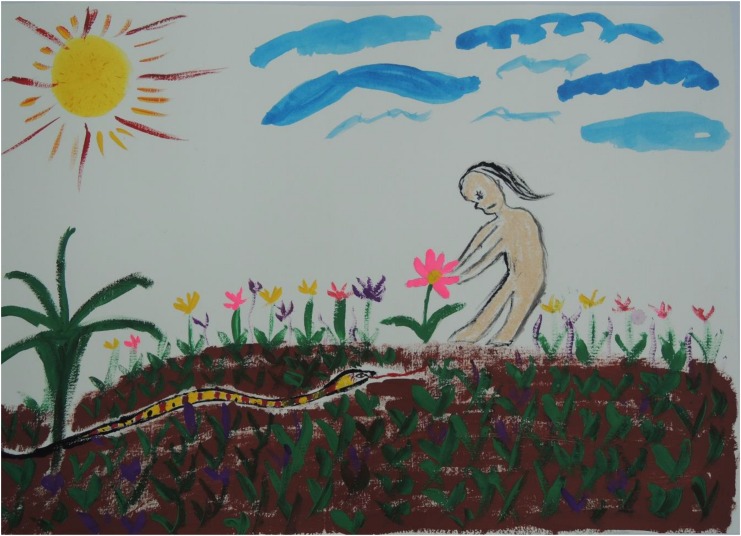
Disgust, painted by a Vietnamese participant.

**FIGURE 8:**
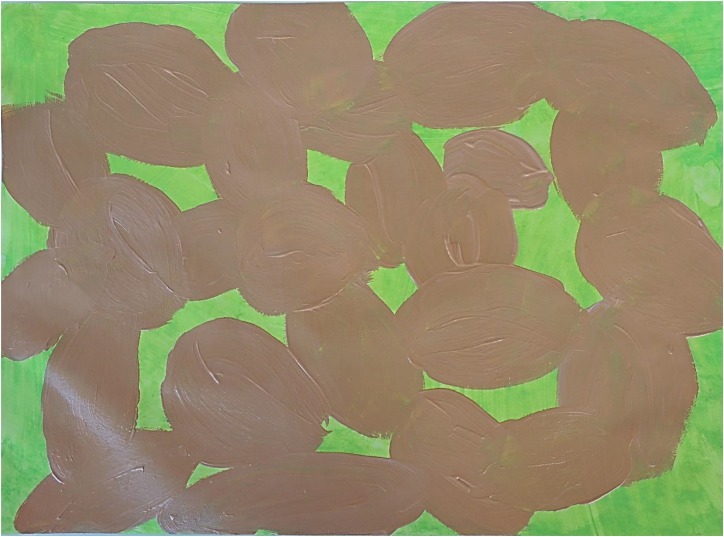
Disgust, painted by a German participant.

Figures 3–8 show some “typical” pictures of Vietnamese participants and “typical” pictures of a German participant. All participants provided written informed consent for the publication of their pictures. The Figures 3, 4 are related to the basic emotion sadness. The German painting is abstract, while the Vietnamese painting is scenic and tells a story. The couple is standing back to back, symbols (flowers) are used to express separation. Figures 5, 6 are related to the emotion of happiness. The Vietnamese painting again is scenic and uses different symbols to tell a story of the experiences of this baby. In contrast, the German painting is much more blurred and expresses happiness by symbolizing the sun in an abstract way. The dots are produced by using golden glitter. Figures 7, 8 are related to the emotion of disgust. The Vietnamese example shows the scenic expression of emotions with partly bright colors and symbols like flowers, sun and snake. The German expression of disgust seems to be related more to bodily disgust; it could remind of feces and is more or less abstract. The colors are brown and green. In all displayed Vietnamese samples human beings are shown.

## Discussion

So, it seems most likely that participants in a Vietnamese art therapy self-experience training create pictures that differ from those of German participants. We found many statistically significant differences. These referred on the one hand to the formal criteria of the art work such as the size of the picture, the number of colors, the type of the painted shapes and the materials that were used. On the other hand, there were significant differences in the content-related criteria such as the expression of the pictures, their concreteness and the use of symbols. The Vietnamese participants preferred smaller formats, mostly used liquid paints and also used more colors than the German participants. In terms of content, the Vietnamese pictures were much more narrative (describing stories), more scenic and concrete, and symbols were used much more frequently.

These differences may be due to many different factors, which even may be linked. In the basic emotions concept by Ekman (1972), it is assumed that these have a physiological cause as well and are experienced by all human beings. At the same time, the emotional expression (linguistic, artistic) significantly depends on the culture of the country, how much emotion is permitted and how it may be displayed.

Lewis et al. (2010) reported on an experiment with preschool children where the facial expressions of Japanese children differed significantly from white and non-white American children when they expressed emotions felt about a success or failure. Röttger-Rössler et al. (2015) showed how children display emotions such as anxiety and fear differently in different countries (Madagascar and Taiwan) due to how they were raised. Morelen and Thomassin (2013) showed in a review article how different the expression of emotion in American groups of different ethnicities is socialized. According to these studies, there are much stronger ties to one’s family in Asian families and an avoidance of negative emotions. Even hierarchies within one’s family, shame and the protection of the family’s honor play an important role. Betts (2013b) reports of a master thesis by Lee (2011) in which paintings of American and Korean children have been compared. The paintings of the Korean children were less colorful, less realistically colored, and smaller and had fewer details than those of the American children.

There are many studies with children about artistic creativity in the context of a standardized task. Often, children are asked to draw their family, for example, or themselves (Rübeling et al., 2011; Gernhardt et al., 2014; Goldner and Levi, 2014). We were unable to find other systematic research in the literature on the artistic creativity of adults in connection with a standardized task as in our study.

Socialization practices in Vietnamese cultures are organized along collectivist dimensions and Confucian philosophy (Dalton et al., 2002; Nguyen, 2016). Like other collectivist societies in Southeast Asia, Vietnamese culture values interdependence and harmonious relationships and give priority to family or communal goals over personal goals. To achieve harmony, a Vietnamese must observe moderation and avoid extremes in conversation, in the day-to-day activities and in all kinds of social interaction. To achieve moderation, Vietnamese people are not encouraged to openly express their emotions. Holistic thinking means that people take into consideration the context when they think about something. Vietnamese culture is collectivistic. It is possible that Vietnamese people consider the context when expressing emotions: Who of the context is involved and what would that mean for the other? This might be reasons, why they prefer to tell stories in their emotion pictures and perhaps display emotions in another way than members of a more individualistic, Western-oriented group.

The results showed that basic emotions such as sadness and disgust were expressed in the pictures with different colors. It is common knowledge that colors in different cultures are also associated with different meanings. A study by Ou et al. (2003) showed, for example, that British and Chinese experience the effect of colors differently. There are also significant differences as to what colors are more popular and which are less popular. The sample was very small, however, with 17 Chinese and 14 British. In advertising as well, a great deal of attention is paid to which colors may be less popular in another culture, for example. In this context, Kommonen (2011) point to the high cultural significance of colors in China. A somewhat older study (Kitao and Kitao, 1986) showed that colors are also associated with linguistic terms and that these differ from one culture to the next (e.g., the red-light district or blue blood).

The learning of artistic abilities also depends on the attitudes of the parents and the school system. A prospective study with several measuring points, in which American children with a Chinese background and American children with a European background were compared, showed that they are introduced to drawing quite differently and that the abilities and creativity differs between the groups as well (Huntsinger et al., 2011).

Another starting point could also be the differences in the development of the approach to mental illnesses and psychotherapy. Here, Vietnam is certainly a country that is still developing and where it is much less common than in Germany to discuss mental phenomena in society and to maybe express them in pictures in art therapy as well (Vuong et al., 2011; Ngo et al., 2014). The development of psychotherapy and art therapy is still very much in its initial stages in Vietnam. In other Asian countries (such as Japan and South Korea) these methods are already much more known and implemented. This is probably true for the personal therapy of psychotherapists as well (Kalmanowitz et al., 2012).

The groups of participants are relatively similar in terms of self-experience regarding their professional experience and their art therapy knowledge. There is, however, one significant difference between the groups: In Germany, only women participated in the training, while in Vietnam, the number of men was much higher than the number of women. This could be another reason that explains the differences described.

Hocoy (2002) discusses to what extent aspects of art therapy, which originated from the European/American region and that emphasize the individuation of the individual, can be transferred to the Asian region with other norms and concepts. He emphasizes here the need for culturally sensitive art therapy. We agree but have the impression that the Vietnamese participants were excited about the idea of expressing emotions in a picture and considered it a good opportunity to do therapeutic work with Vietnamese clients and patients as well. We assume that, due to their culture, it might be somewhat easier for Vietnamese to express emotions in a picture than to talk about them.

### Limitations

The study was designed as an explorative study, and the results cannot claim to be representative. The groups were rather small and heterogeneous. In Germany, the groups consisted of young art therapy students and in Vietnam mostly of physicians and psychologists, who are interested in art therapy and want to learn more about it. The rating method that was used focuses on formal and content-related aspects but does not document any possible interpretations of the pictures or the verbal comments made by the training participants or the therapist. The interrater correlation for the documented criteria was, however, quite good. For therapeutic reasons, there were group discussions after the paintings had been completed. This fact might also have influenced the way of painting in the next session.

### Outlook

In a subsequent study, we will address the opinions of the training participants about art therapy and its prospects in Vietnam. To this purpose, interviews were held with the training participants in Vietnam, which have not yet been analyzed, however. We plan to continue to support the development of art therapy in Vietnam and to support it through scientific research as well. A next question would be, for example, whether the differences that we found in the training participants can be shown in treated patients in a similar form.

## Author Contributions

AD-W is the head art therapist in this study, performed the workshops in Germany and Vietnam, and collected the data. KP, MK, and JB performed the ratings of paintings. MT, TL, and LN organized the workshops in Vietnam and contributed mainly to the discussion about intercultural differences. NK organized the German workshops and the German data assessment. ER performed the data analysis and statistics. HG, as the head of the department, was actively involved in developing, planning, and discussing the study. JvW together with AD-W (the Principal Investigator) wrote the manuscript. All authors were actively involved in the discussion of the manuscript.

## Conflict of Interest Statement

The authors declare that the research was conducted in the absence of any commercial or financial relationships that could be construed as a potential conflict of interest.
